# Safe and effective delivery of supplemental iron to healthy older adults: The double-blind, randomized, placebo-controlled trial protocol of the Safe Iron Study

**DOI:** 10.12688/gatesopenres.13039.2

**Published:** 2021-02-09

**Authors:** Erin D. Lewis, Dayong Wu, Joel B. Mason, Athar H. Chishti, John M. Leong, Kathryn Barger, Simin N. Meydani, Gerald F. Combs

**Affiliations:** 1 Jean Mayer USDA Human Nutrition Research Center on Aging, Tufts University, Boston, Massachusetts, 02111, USA; 2 Department of Developmental, Molecular and Chemical Biology, Tufts University, Boston, Massachusetts, 02111, USA; 3 Department of Molecular Biology and Microbiology, Tufts University, Boston, Massachusetts, 02111, USA

**Keywords:** Iron supplementation, iron-replete, ferrous sulfate, malaria, bacterial proliferation, inflammation

## Abstract

The forms of iron currently available to correct iron deficiency have adverse effects, including infectious diarrhea, increased susceptibility to malaria, inflammation and detrimental changes to the gut microbiome. These adverse effects limit their use such that the growing burden of iron deficiency has not abated in recent decades. Here, we summarize the protocol of the “Safe Iron Study”, the first clinical study examining the safety and efficacy of novel forms of iron in healthy, iron-replete adults. The Safe Iron Study is a double-blind, randomized, placebo-controlled trial conducted in Boston, MA, USA. This study compares ferrous sulfate heptahydrate (FeSO _4_·H _2_O) with two novel forms of iron supplements (iron hydroxide adipate tartrate (IHAT) and organic fungal iron metabolite (Aspiron™ Natural Koji Iron)). In Phase I, we will compare each source of iron administrated at a low dose (60 mg Fe/day). We will also determine the effect of FeSO _4 _co-administrated with a multiple micronutrient powder and weekly administration of FeSO _4_. The forms of iron found to produce no adverse effects, or adverse effects no greater than FeSO _4_ in Phase I, Phase II will evaluate a higher, i.e., a therapeutic dose (120 mg Fe/day). The primary outcomes of this study include *ex vivo *malaria ( *Plasmodium falciparum*) infectivity of host erythrocytes, *ex vivo *bacterial proliferation (of selected species) in presence of host plasma and intestinal inflammation assessed by fecal calprotectin. This study will test the hypotheses that the novel forms of iron, administered at equivalent doses to FeSO _4_, will produce similar increases in iron status in iron-replete subjects, yet lower increases in *ex vivo* malaria infectivity, *ex vivo* bacterial proliferation, gut inflammation. Ultimately, this study seeks to contribute to development of safe and effective forms of supplemental iron to address the global burden of iron deficiency and anemia.

**Registration:** ClinicalTrials.gov identifier: NCT03212677; registered: 11 July 2017.

## Introduction

The forms of iron currently available have adverse effects that limit their use in addressing prevalent iron deficiency. Iron-deficiency, and the anemia it can produce, affects some 1.2 billion people worldwide, mostly children and women in resource-poor countries, comprising a global disease burden that has not abated in recent decades. Efforts to address iron deficiency through iron supplementation programs have been frustrated by the serious side effects of inorganic forms of iron, which due to lower enteric absorptive efficiency compared to heme-iron in foods (2–20%), must be given in relatively high levels ^1^. Those adverse effects include infectious diarrhea ^2– 5^, detrimental changes in the gut microbiome ^5, 6^ and increased serious morbidity among iron-replete children in malaria-endemic areas ^7, 8^, as well as less serious consequences such as dyspepsia and constipation. One of the underlying causes of these effects are thought to involve stress on the gut due to unabsorbed iron, which can be pro-oxidative and pro-inflammatory. In addition, unabsorbed, soluble iron can be used by the gut microbiome and favor the proliferation of pathogenic enteric bacteria ^9^, which can contribute to the inflammatory response ^5, 6, 10, 11^ that leads to down-regulation of iron absorption ^12^. Further, the formation of non-transferrin-bound iron (NTBI) from the high supplemental sources of iron is believed to be involved in the increase of malaria infection severity ^13, 14^. In capillaries of the intestine and brain, NTBI is proposed to increase sequestration of erythrocytes infected with malaria ^15^.

The serious adverse effects of current iron supplements are problematic because they have deterred addressing prevalent iron deficiency in regions of need. Specifically, the finding that routine iron supplementation led to increased severity of clinical malaria among children in Tanzania ^7^ effectively halted efforts to address iron deficiency in malaria-endemic areas. Lack of a safe and effective treatment leaves large numbers of children iron deficient, many with associated anemia. Thus, the burden of disease, which includes growth, cognitive and physical performance deficits as well as increased risk of infection, continues to climb in this age group.

### Rationale

The overall goal of this project is to generate evidence needed for the development of a modality of providing bioavailable iron that minimizes or eliminates adverse effects in iron-replete individuals. We will employ the commonly used iron supplement, ferrous sulfate (FeSO _4_) to compare with two novel forms of iron with features that suggest each may be a useful nutritional source of iron with fewer side effects than FeSO _4_.

The first novel form of iron is a nanoparticulate formulation of iron hydroxide adipate tartrate (IHAT), which was developed and donated to this study by the Medical Research Council, Cambridge University and, currently, Nemysis Ltd. IHAT is a nanoparticle-based supplement composed of three generally recognized as safe (GRAS) substances, iron hydroxide, tartaric acid, and adipic acid. The particle itself resembles the normal metabolite ferritin, which is a larger polyatomic particle. Like ferritin, IHAT can be absorbed by endocytosis, but dissociates within the enterocyte and is subsequently metabolized as ferrous iron. In pre-menopausal, iron-deficient women, the relative bioavailability of IHAT to FeSO _4_ was approximately 75% ^16^. Once absorbed, IHAT appears to enter the metabolic iron pool at efficiencies comparable to iron from FeSO _4_, and it is insoluble in the lumen of the gut ^16, 17^. That appears to prevent its being utilized by enteric bacteria, as evidenced by the finding that it did not affect the recovery of the hindgut microbiome in iron-deficient mice ^18^. Thus, we anticipate that it is likely to be less irritating to the gut and, likely, less pro-inflammatory than FeSO _4_. In addition, IHAT appears to enter the metabolic iron pool more slowly than iron from FeSO _4_^19^, suggesting that its post absorptive surge in pro-oxidative NTBI may be less than that of FeSO _4_, thus, producing less oxidative stress.

The second novel form of iron is an organic fungal iron metabolite, Aspiron™ Natural Koji Iron, which has been developed and donated to this study by Cura Global Health Inc. Aspiron is a product of the natural fermentation of Koji ( *Aspergillus oryzae)* in the presence of FeSO _4_. The iron-rich Koji biomass is heated, harvested and dried resulting in the inactivation of Koji powder that contains 8–10% iron. Koji ( *A. oryzae)* is widely used for making such foods as soy sauce, tempeh, miso, and for producing food-grade α-amylase. Koji is considered safe by Joint FAO/WHO Committee on Food Additives and has been accepted as a GRAS constituent of food. The Codex Alimentarius International standards included Koji as an approved food additive. In non-pregnant, non-anemic women with marginal iron stores (ferritin concentration of <40 μg/L), the absorption of Aspiron was comparable to FeSO _4_, suggesting similar tolerability as FeSO _4_^20^. The kinetics of Aspiron absorption shows it to appear in the plasma later, and over a longer period of time, than iron in FeSO _4_. Therefore, we anticipate that any post absorptive surge in pro-oxidative transferrin-iron and NTBI will be less than that of FeSO _4_, thus, also producing less oxidative stress. Moreover, Aspiron was found to have significantly lower number of side effects compared to FeSO _4_^20^. 

This study is divided to two phases. In Phase I, we will determine effects of both forms of low-dose supplemental iron. For the forms of iron found to produce no adverse effects at the 60 mg Fe/day dose level, Phase II of the study will be conducted in which such forms will be tested at a higher, therapeutic dose of 120 mg Fe/day against the same outcomes.

Here, we summarize the protocol for the Safe Iron Study, the first clinical study examining the safety and efficacy of these novel forms of iron supplements in iron-replete older adults.

### Hypothesis and objectives

We will robustly test our formal hypotheses yielding useful answers to the primary questions about the relative safety and efficacy of these novel forms of iron in iron-replete subjects. This study will address the major side effects of oral iron supplementation of iron-replete subjects. Specifically, we will compare the effects of two novel forms of iron and the widely used ferrous form on malaria ( *Plasmodium falciparum*) infectivity of host erythrocytes (assessed *ex vivo*), bacterial proliferation (of selected species) in the presence of host plasma (assessed *ex vivo*), redox stress and inflammatory stress (assessed *in vivo*), gut irritation (assessed *in vivo*), effects on the gut microbiome (assessed *in vivo*). This set of indicators is referred to as the “safety profile”. Additionally, iron utilization (assessed *in vivo*) will be compared to evaluate efficacy.

In Phase I, we will test the following research questions underpinning formal hypotheses using the design detailed below:

1. Does IHAT, a novel form of iron, produce beneficial and/or adverse effects comparable to FeSO _4_ at equivalent iron doses?

IHAT and FeSO _4_ administered at 60 mg Fe/day will produce comparable responses in malaria infectivity, bacterial proliferation, and iron status parameters. However, IHAT will produce less gut/systemic inflammation, and fewer effects on the microbiome.

2. Does Asprion, a novel form of iron, produce beneficial and/or adverse effects comparable to FeSO _4_ at equivalent iron doses?

Aspiron and FeSO _4_ administered at 60 mg Fe/day will produce comparable responses in malaria infectivity, bacterial proliferation, and iron status parameters. However, Aspiron will produce less gut/systemic inflammation, and fewer effects on the microbiome.

3. Are the adverse effects of FeSO _4_ affected by co-administration of a multi-micronutrient powder (MNP)?

The safety profile of FeSO _4_ for iron-replete individuals is not affected by its administration with a multiple micronutrient powder.

4. Are the beneficial and/or adverse effects of FeSO _4 _comparable when administered daily or weekly?

The timing of FeSO _4_ administration will not affect malaria infectivity, bacterial proliferation, and iron status parameters; however, weekly administration will produce less gut/ systemic inflammation and fewer effects on the microbiome.

In Phase II, we will test the following formal hypotheses using the design detailed below:

1. Does IHAT, a novel form of iron, effect positive improvements in iron status comparable to FeSO _4_ at equivalent iron doses, while producing fewer adverse effects?

IHAT and FeSO _4 _administered at 120 mg Fe/day will produce similar increases impacting iron status in Fe-replete subjects. However, IHAT will produce relatively lower increases in *ex vivo* malaria infectivity, *ex vivo* bacterial proliferation, redox stress, gut/systemic inflammation, and fewer effects on the hindgut microbiome.

2. Does Aspiron, a novel form of iron, effect improvements in iron status comparable to FeSO _4_ at equivalent iron doses while producing fewer adverse effects?

Aspiron and FeSO _4 _administered at 120 mg Fe/day will produce similar increases on iron status in Fe-replete subjects. However, Aspiron will produce relatively lower increases in the *ex vivo* malaria infectivity, *ex vivo* bacterial proliferation, redox stress, gut/systemic inflammation, and fewer effects on the hindgut microbiome.

### Study design

The Safe Iron Study is a double-blind, randomized, placebo-controlled study with iron supplementation in iron-replete older adults. For Phase I, adults are randomized to one of six intervention groups ( Table 1). For Phase II, adults are randomized to one of three intervention groups ( Table 2). For both phases, each arm undergoes an intervention period of 4 weeks, after which changes in parameters will be compared between baseline (week 0) and post-intervention (week 4) study visits.

**Table 1.** Intervention groups for Phase I of the Safe Iron Study.

Subjects	Intervention Group	N	Intervention	Sampling
Fe-replete, PMW and men	Intervention 1	25	Placebo	0 and 4 weeks of intervention
	Intervention 2	25	FeSO _4_ (60 mg Fe/day)	0 and 4 weeks of intervention
	Intervention 3	25	FeSO _4_ (420 mg Fe/week)	0 and 4 weeks of intervention
	Intervention 4	25	FeSO _4_ (60 mg Fe/day) + MNP	0 and 4 weeks of intervention
	Intervention 5	25	IHAT (60 mg Fe/day)	0 and 4 weeks of intervention
	Intervention 6	25	Aspiron (60 mg Fe/day)	0 and 4 weeks of intervention

PMW, post-menopausal; FeSO _4_, ferrous sulfate; MNP, multiple micronutrient powder; IHAT, iron hydroxide adipate tartrate

**Table 2.** Intervention groups for Phase II of the Safe Iron Study.

Subjects	Intervention Group	N	Intervention	Sampling
Fe-replete, PMW and men	Intervention 1	25	FeSO _4_ (120 mg Fe/d)	0 and 4 weeks of intervention
	Intervention 2	25	IHAT (120 mg Fe/d)	0 and 4 weeks of intervention
	Intervention 3	25	Aspiron (120 mg Fe/d)	0 and 4 weeks of intervention

PMW, post-menopausal; FeSO _4_, ferrous sulfate; IHAT, iron hydroxide adipate tartrate

## Methods: Subjects, interventions and outcomes

### Study setting

The majority of the research will be conducted at the Jean Mayer United States Department of Agriculture (USDA) Human Nutrition Research Center on Aging (HNRCA), Boston, MA, USA. The HNRCA has in-house recruiting and human subject management teams, medical oversight, and a wide spectrum of statistical, biochemical and molecular biological analytical expertise. The *ex vivo P. falciparum* infectivity and bacterial proliferation studies will be conducted at the Tufts University Graduate School of Biomedical Sciences, Boston, MA, USA.

### Eligibility criteria

Subjects are healthy older adults from Boston and surrounding area and must meet all of the inclusion criteria and none of the exclusion criteria to be considered eligible for the study.

***Inclusion criteria.*** Apparently healthy men and post-menopausal women (defined as no menses for ≥1year); between 50–80 years of age; BMI range between 18–35 kg/m ^2^; typical bowel pattern of at least one stool every other day; willing to comply with study procedures; and will not be undergoing colonoscopy in the 2 months before, or during the course of the study.

***Exclusion criteria.*** Any major illness or condition that may interfere with study outcomes at the discretion of the study physician; personal history of glucose-6-phosphate dehydrogenase deficiency; type 1 or type 2 diabetes, or use of any pharmacological treatment for diabetes; endocrine disorders including unstable thyroid disease (dose adjustment of thyroid replacement in the past 6 months), adrenal disease, pheochromocytoma or parathyroid disease; recent history of inflammatory diseases; use of TNF blockade medication, methotrexate, or other immune-modulating drugs; steroid use (except for non-prescription topical and nasal steroids); change in dose regiment in past month or expected change during course of study if using hormone replacement therapy with estrogen, testosterone or growth hormone; history of myocardial infarction, stroke or total ischemic attack, coronary artery bypass graft, stenosis >50% diagnosed within the past year or acute unstable cardiovascular disease; clotting/bleeding disorders or ongoing anticoagulant use; gastrointestinal (GI) diseases, conditions or medications known to influence GI absorption; history of stomach or bowel resection (other than appendectomy), gastric bypass or other bariatric weight loss procedure; regular use (>2 times per week) of acid lowering medication; history of eating disorder anorexia, bulimia or binge-eating in the past 5 years; actively undergoing dialysis; inadequately controlled hypertension, certain psychiatric disorders; immunodeficiency condition, HIV or AIDS; cancer of any type (except for non-melanoma skin) or cancer therapy in past year; actively using cancer chemotherapeutic agents; regular use of ASA, NSAIDs or Cox-2 inhibitors; infection or febrile illness within two weeks prior to study or study blood draws; history, vaccination or treatment for malaria, or antimalarial prophylaxis in past 3 months; vaccinations (excluding malaria vaccine) <72 hours prior to all blood draws; unmanaged seizure disorders; history of splenectomy; chronic liver disease; use of fiber supplements, laxatives or stool softeners; colonoscopy procedure or prep within 2 months prior to or during study; predicted or anticipated antibiotic use within 3 months prior to or during study participation; unwilling to maintain dose regimen during study if using probiotic or prebiotic; inability to deliver stool sample within 18 hours of bowel movement; alcohol use on average >2 servings/day or >14 servings/week or self-reported binge drinking; current use of iron; unwilling to stop all dietary supplement, vitamins (except calcium or vitamin D), minerals, herbal other plant-based preparations, fish oil supplements or homeopathic remedies use at least one month prior to study; inadequate venous access or history of a bilateral mastectomy with nodal dissection; participation in other research study during the same time period; no social security number; iron saturation outside of normal range; hemoglobin <11.7 (females) <13.2 (males); serum creatinine >1.5 mg/dl; fasting blood sugar ≥126 mg/dL; SGOT >1.5 x upper range of normal; SGPT >1.5X upper range of normal mg/dL in absence of benign cause.

### Interventions

This study will be using two novel forms of iron (IHAT and Aspiron) compared to FeSO _4_. One arm will also include an MNP (MixMe™ Vitamin and Mineral Powder) donated by DSM Nutritional Products (Switzerland). The placebo will be comprised of Melojel® corn starch, donated by Ingredion (Manitoba, Canada). All forms of iron, MNP and corn starch have been approved for use in humans. This trial is only intended to evaluate the different supplement forms of iron on the structure or function of the body, not for a therapeutic purpose. All raw materials will be sent to the Richardson Centre for Functional Foods and Nutraceutical (RCCFN) at the University of Manitoba, Winnipeg, Manitoba, Canada for encapsulation. All interventions will be encapsulated into opaque, double zero capsules and appear identical in size, color and weight. The RCCFN is licensed as a Health Canada Natural Health Product Site with the ability to produce good manufacturing practices (GMP) and Hazard Analysis and Critical Control Points (HACCP) certified products. For the administration of capsules, each subject will receive a number-coded calendar-style monthly pill planner with a set number of capsules.

***Adherence.*** Adherence to supplement use will be assessed by using pill counts, a compliance calendar and iron status. Pill counts will be based on the number of pills remaining in the returned monthly pill planner. Subjects will be sent home with a compliance calendar to record their supplement intake and will be instructed to bring this document with them on the post-intervention study visit. Subjects will be instructed to return the number-coded calendar-style monthly pill planner with any unused sections or pills at the end of the 4-week study visit. The number of unused pills will be recorded at the 4-week study visit. Parameters of iron status will be measured in serum and will serve as additional measures of adherence to supplement protocol.

### Outcomes

The Safe Iron Study has three primary outcomes to test the formal hypotheses in Phases I and II. These outcomes include malaria ( *Plasmodium falciparum*) infectivity of host erythrocytes (assessed *in vitro*), bacterial proliferation (of selected species *Staphylococcus aureus*, *Escherichia coli*, *Acinetobacter baumannii*, *Klebsiella pneumoniae* and *Salmonella typhimurium*) in presence of host plasma (assessed *ex vivo*) and intestinal inflammation assessed by fecal calprotectin concentrations. Secondary outcomes include iron status parameters (hemoglobin, transferrin, ferritin, serum iron, total iron-binding capacity (TIBC), percent saturation, NTBI, and fecal iron), assessments of systemic inflammation (CBC/diff, *ex vivo* cytokine production, *in vivo* inflammatory cytokines), additional assessments of intestinal inflammation (fecal neopterin, myeloperoxidase, alpha-1 antitrypsin, cytokines, fecal immunochemical test (FIT) and plasma tryptophan and kynurenine), alterations in gut microbiome (microbiota analysis and short chain fatty acids) and redox status (F2α-isoprostanes and glutathione). A detailed outcome measurement plan is included in Table 3.

**Table 3.** Timing and analytical details of questionnaires, biological and chemical analyses conducted at each study visit.

Time of Sampling							Specimen			
Screen	Baseline (0 wks)		Post- Intervention (4 wks)		Intra Study (0–4 wks)		Whole Blood	Serum	Plasma	Stool
Fast	Fast	2 hrs	Fast	2 hrs						
+	+		+			General Health/ Medical History				
+						Diet History				
					+	24-h Dietary Recalls				
+	+		+		+	Gut Irritation Questionnaire				
+						Standard Health Screening Questionnaire				
	+			+		Malarial Infectivity	*In vitro*			
	+	+	+	+		Bacterial Proliferation		Assessed *in vitro*		
+						Health Status		Chem Profile		
+	+		+			Systemic Inflammation	CBC/diff			
	+		+				*Ex vivo* cytokine production			
	+	+	+	+					*In vivo* inflammatory cytokines	
	+		+			GI Inflammation				Fecal immune Chem Test (FIT)
	+		+						Tryptophan, Kyneurenine	Calprotectin, Neopterin, Myeloperoxidase, Cytokines, α1- antitrypsin
	+		+			Gut Microbiome				Fecal SCFAs
	+		+							Microbiota DNA Analysis
	+	+	+	+		Redox Stress			F2α- isoprostanes, GSH/GSSG	
+	+	+	+	+		Fe Status	Hb	Fe, Transferrin, Transferrin saturation		
	+	+	+	+				Ferritin, Transferrin receptor, Hepcidin		
	+	+	+	+				Non-Transferrin- bound-Fe (NTBI)		

### Recruitment and screening

Subjects are recruited from the general public in the greater Boston area using several recruitment strategies including local print and electronic media (such as advertisements in newspapers, craigslist, local newsletters, bulletin boards, different websites and radio) as well as posting flyers in public places such as local YMCAs, supermarkets, libraries, laundromats, local community organizations and health centers. Additionally, a roster of >20,000 names of previous study subjects from which potentially qualifying women and men within the specified age and BMI can be identified and contacted through the use of direct mailings.

Prospective subjects will provide oral consent form prior to completing a telephone pre-screening questionnaire, and if they appear to be qualified, they will be invited to the HNRCA for screening. This will allow for the recruitment of only those who are confident they are able to complete all study activities. The full screening process will be conducted in a quiet location by an experienced research study nurse at the Metabolic Research Unit (MRU) at the HNRCA, and those interested will be asked to sign the screening consent form (Supplementary File 1, available as *Extended data* ^21^) prior to the start of all screening procedures. Subjects will then undergo blood tests and a review of their medical history and general health including gastrointestinal health history, gut irritation questionnaire, use of medications and nutritional supplements including iron. Once an individual has gone through the screening process and is deemed eligible to participate, they will be informed by telephone by a staff at the MRU that they are eligible and if they still agree to participate, they will be scheduled to come to the HNRCA for the enrollment and baseline study visits.

### Study timeline and visits

**Figure 1.**
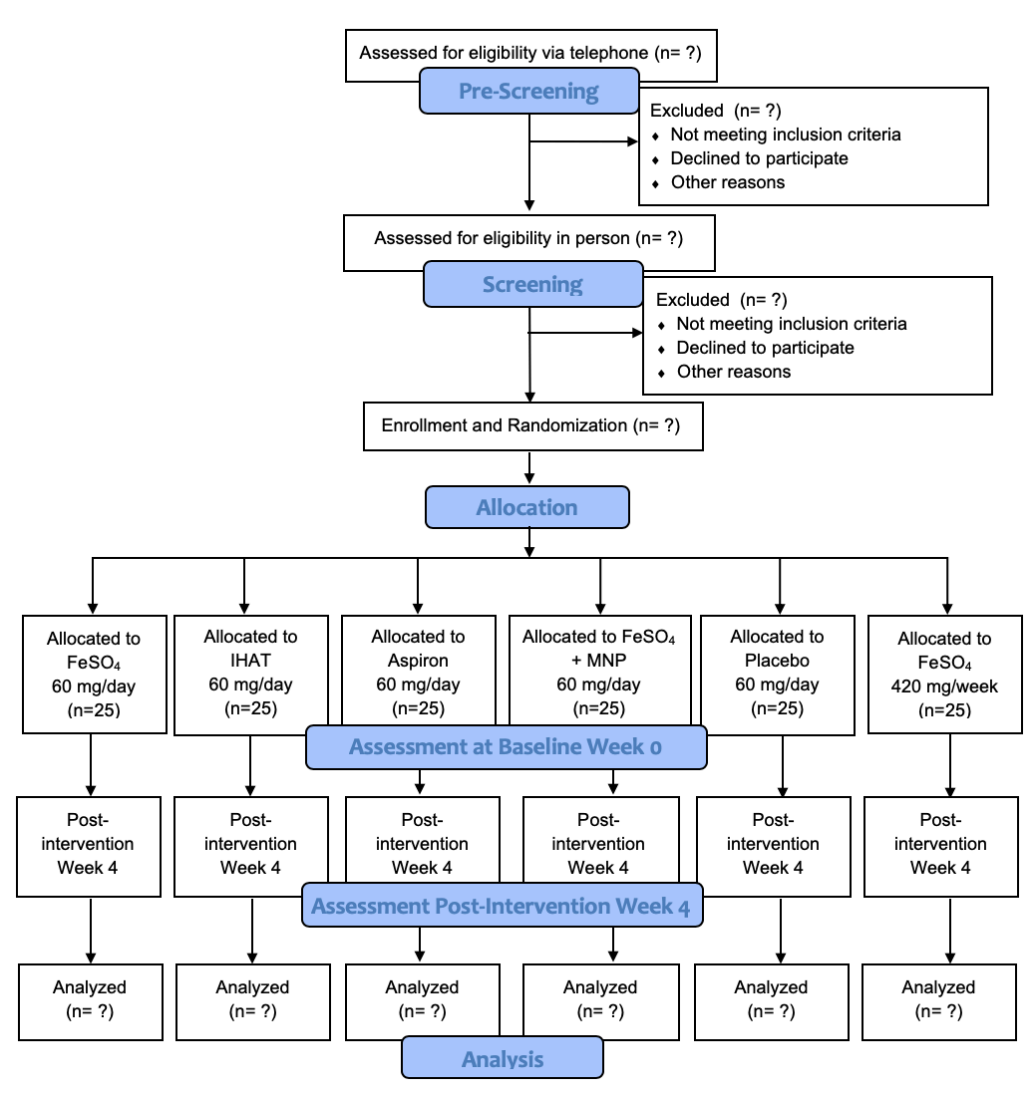
Phase I CONSORT flow chart.

**Figure 2.**
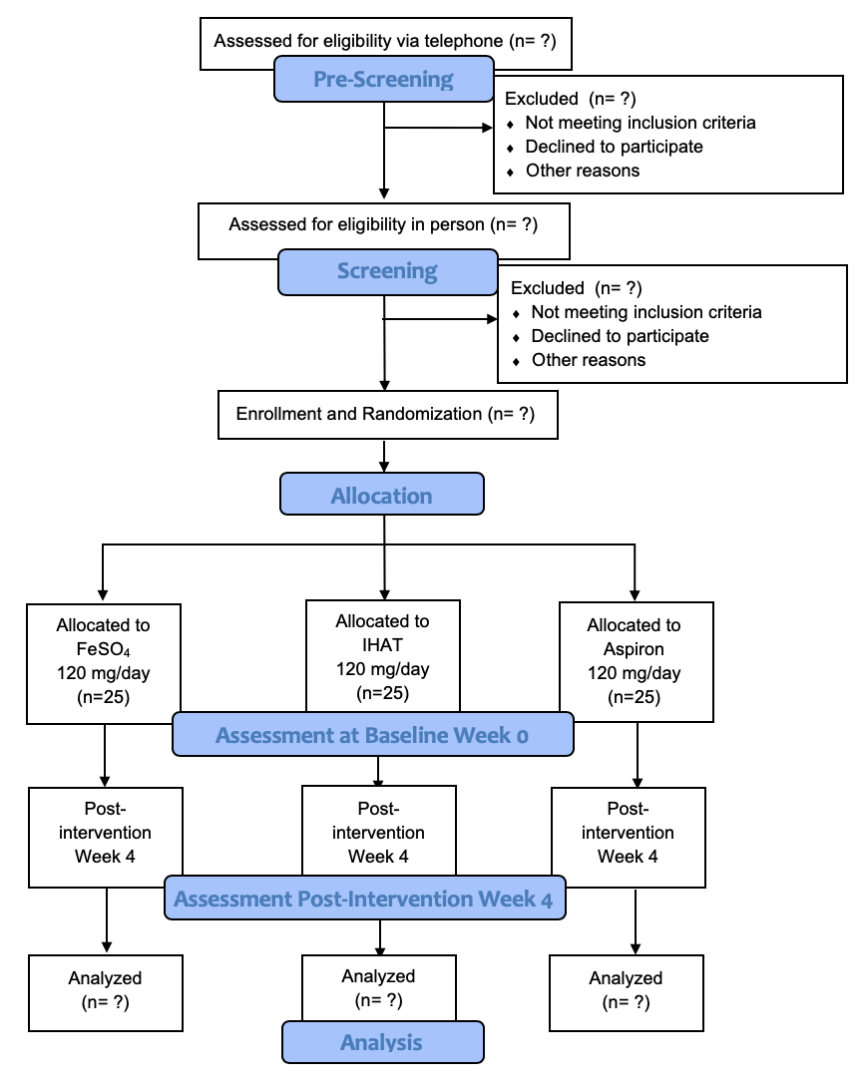
Phase II CONSORT flow chart.

Illustrated in Figure 1 (Phase I) and Figure 2 (Phase II), eligible subjects will be identified using pre-screening and screening procedures, as described above. Once an eligible subject is identified and wishes to participate, they will complete an enrollment visit for which informed consent (Supplementary File 2, available as *Extended data* ^21^) is obtained and instructions for stool sample collection are reviewed. The eligible subject is then randomized to a treatment group. The subject returns for a baseline, pre-intervention visit (week 0) for which a stool sample, fasted blood sample and questionnaire assessing signs and symptoms of gastrointestinal health (Gut Irritation Questionnaire) are collected, followed by standard continental breakfast (English muffin or bagel, spreads, coffee, juice and fruit). Oranges (juice, marmalade, or fresh fruit), and teas will not be offered, as those foods decrease the absorption of iron in the body. Immediately following breakfast, subjects will be administered the assigned intervention agent. After 2 hours, a post-prandial, post-dose, blood sample is collected. Instructions for intervention agents are reviewed with the subjects and they are dispended study supplements sufficient for 4 weeks. During the 4-week intervention period, subjects complete weekly Gut Irritation Questionnaires in addition to three 24-hour dietary recalls via telephone. After the 4-week intervention, subjects return for a post-intervention visit which follows the same procedures as the baseline visit.

## Methods: Assignment of interventions

### Randomization and blinding

The allocation sequence constructed using a block randomization with a block size of four. Random ordering of the allocations within each block was performed using for the study was performed using SFMT (SIMD-oriented Fast Mersenne Twister) algorithm (version 1.5.1) based randomization functions in the “StatsBase” Julia package. No stratification was used in the randomization, and the allocation sequence was implemented using the built-in “randomization” module in REDCap. This module references the provided allocation table and assigns participants based on order of enrollment.

For Phase I, subjects will be randomly assigned to one of the six treatment arms outlined in Figure 1 at the time they enroll in the study. A randomization scheme for all entering subjects will be generated and stored by the Data Manager. The randomization sequence is known only to the Data Manager, and REDCap user roles were used to prohibit access to the allocation sequence until the participant was randomized to a treatment group. The unblinded key is maintained by the data manager and stored in a locked filing cabinet until the data collection for the study is complete. An active control group, using a corn starch placebo, will be used to provide a control for prospective changes over time and mimic any effects of ingesting a daily capsule in case it may be relevant. We will also provide placebo capsule to all other groups to blind against the FeSO _4_ plus MNP group. All daily interventions groups will receive three physically similar capsules to ensure blinding of the treatments. The weekly intervention group will receive four physically similar capsules once per week.

For Phase II, subjects will be randomly assigned to one of the three treatment arms outlined in Figure 2 at the time they enroll in the study. The randomization scheme will be the same as Phase I. We will not employ a control group in Phase II because any prospective changes over time or effects of ingesting a daily capsule will be determined from Phase I. All groups will receive four physically similar capsules to ensure blinding of the treatments.

### Withdrawal of subjects

Subject participation may be terminated should they no longer meet any of the study criteria cited above, or if they fail to comply with study requirements or if a reported adverse reaction occurs that is considered severe. Subjects are free to withdraw from participation at any time during the study by writing, calling or emailing the study PI. The decision to terminate a participant from the study will be based on the judgment of the study physician and the study PI. If a participant does not complete the entire study, their stipend will be prorated accordingly depending on where the participant is in the study prior to withdrawal. If a subject withdraws or is withdrawn from the study, any data or specimens collected before withdrawal will still be used in the study.

## Methods: Data collection, management and analysis

### Data collection methods

***Laboratory analyses.*** At baseline (week 0) and post-intervention (week 4), subjects will provide blood and stool samples as well as complete several questionnaires. Blood pressure and pulse rates as well as anthropometric measurements will also be taken. Table 3 outlines the questionnaires, biological and chemical analyses conducted at each study visit. Details of the analyses are described below.

***Blood samples.*** Blood samples will be collected at baseline and post-intervention. Depending on the analyses, 12-hour fasted and 2-hour postprandial, post-supplementation blood samples will be collected ( Table 3) and the following parameters will be assessed: CBC/differential; *P. falciparum* infectivity of erythrocytes will be determined *ex vivo* ^22^; bacterial proliferation potential will be conducted *ex vivo* on plasma ^23^; plasma cytokines and pro-inflammatory markers (Meso Scale Discovery, MSD); *ex vivo* mitogen or antigen-induced cytokine production) commercial ELISAs, R&D Systems, Minneapolis, MN ^24, 25^); iron and unsaturated iron-binding capacity (AU400 clinical chemistry analyzer, Beckman Coulter, Inc., Brea, CA); serum ferritin (commercial human ferritin ELISA, Thermo Scientific, Frederick, MD); transferrin (immunoturbidimetric procedure using the AU400 clinical chemistry analyzer, Beckman Coulter); transferrin saturation (determined from the relationship of the iron to total iron-binding capacity); transferrin receptor (commercial ELISA, R&D Systems); serum NTBI (serum treated with *tris*-carbonatocobalate(III) trihydrate to saturate non-liganded transferrin binding sites followed by precipitation of proteins and ultrafiltration. Iron in the ultrafiltrate will be determined by electrothermal atomic absorption spectrophotometry or mass spectrometry (MS); hepcidin (commercial competitive ELISA, Bachem Group, Torrance, CA ^26^); tryptophan and kynurenine (liquid chromatography (LC)/MS/MS ^27^; F2α-isoprostanes (in-line solid phase extraction prior to quantitative analysis by ultra-performance LC/MS/MS ^28^); reduced glutathione/oxidized glutathione (Hypercarb HPLC column and quantitative detected by LC/MS/MS ^29^).

***Stool samples.*** Subjects will be asked to provide a sample at baseline and post-intervention visits Subjects will receive stool sample collection kit at the enrollment visit to bring the samples to study visits. The stool sample in the collection container will be placed in a plastic bag, surrounded by frozen gel packs to keep it cool for up to 18 hours. Aliquots of homogenized stool will be created and the following parameters will be assessed: fecal immunochemical tests (FIT) (OC-Auto Micro 80 Analyzer); calprotectin (commercial ELISA, R&D Systems, ^30^); myeloperoxidase (commercial ELISA, R&D Systems); neopterin (competitive ELISA, ALPCO, Salem, NH); inflammatory cytokines (Meso Scale Discovery, MSD); alpha-1 antitrypsin (commercial ELISA, R&D Systems); short chain fatty acids (derivatized and analyzed by LC-MS ^31^); microbiome DNA sequencing (stool DNA extracted using the QIAamp DNA Stool Mini kit, Qiagen, Valencia, CA, and amplicons of the V4 region of the bacterial 16S ribosomal DNA will be generated using primers ^32^. Amplicons will be purified on Angencourt AMpure XP beads (Beckman Coulter, Brea, CA) before submission to the Tufts University Core Facility for 250 bp paired-end sequencing on an Illumina MiSeq. Marker-gene sequencing data will be processed using the Quantitative Insights Into Microbial Ecology (QIIME) package ^33^.

### Questionnaires

***Gut irritation questionnaire.*** This questionnaire ^34^ is designed to be self-administered but subjects will be given the opportunity to ask questions about how to fill out the questionnaire. This questionnaire is slightly modified from a validated questionnaire designed to address symptoms of gastrointestinal (GI) irritation after FeSO _4_ supplementation ^34^. It will be completed at screening, baseline (week 0) and post-intervention study visits. Subjects will be given a copy of the questionnaire to take home. Subjects will be administered this questionnaire during weekly phone calls in which they will use to copy of the questionnaire to follow along with questions as they are asked over the telephone.

***Dietary assessment.*** Study subjects will receive a Food Amount Booklet and information about the 24-hour recalls at the baseline visit (Day 0). Three separate 24-hour recalls will be collected by phone during the 4-week study. Dietary intake data will be analyzed using Nutrition Data System for Research (NDSR) software version 2016, developed by the Nutrition Coordinating Center (NCC), University of Minnesota, Minneapolis, MN. Recalls will be collected using the multiple-pass 24-h recall method, assisted by the NDSR software and the goal for each participant will be to collect two weekdays and one weekend day of intake. Outcome data from NDSR will include daily estimated energy and nutrient intake, as well as food, food group, and dietary supplement data.

### Data capture and management

The research data for this clinical trial will mainly consist of questionnaires and clinical measures in whole blood, serum, plasma and stool related to the proposed interventions. These data will be captured and managed using a REDCap database designed specifically for this study using the HNRCA’s deployment of REDCap which are both HIPAA and 21CFR11 compliant. Any data imported into the REDCap database will undergo external validation checks to ensure that the data have been imported correctly. Additionally, metadata files and data dictionaries/codebooks will be used to provide all information necessary to properly use and understand the data files. The HNRCA data management program records and stores subject identifiers separately from clinical information and results of study analyses. A study code number will identify each laboratory specimen and the master list which links the identity of the subject with the laboratory specimens will be kept in a locked file cabinet in the office of the PI and on a password-protected database on a secure Tufts server. The study team will design a tracking and data management system that will include features to improve data quality such as double data entry and edit resolution to ensure that the study variables accurately represent data on the study data collection forms. There will be a tracking and data-management system, trouble shooting for any data entry problems, an audit trail of database editing and range checks for cleaning, daily backup, cleaning of transitional databases, and transfer of master databases to SAS or SPSS for analysis by the study statistician.

### Data access

Access to the study database will be restricted to only the forms relevant for each investigator until completion of the study and the unblinding of the investigators. Additionally, protected health information will automatically be de-identified for all viewing and data exports. The data analyst will be responsible for managing the user access groups and masking all non-relevant information from the investigators.

### Audit trail and backups

A complete audit history for all data and data collection forms will be maintained using the REDCap audit feature, which records every action performed by each user while in REDCap. The study database will be subject to daily backups to tape array and daily, weekly and monthly backup copies will be maintained by Scientific Computing at the HNRCA. After key points in data acquisition, clean up and analysis, additional an additional off-site copy of the data will be created and stored within an electronic laboratory notebook using the LabArchives electronic laboratory notebook software. 

### Statistical methods

***Summary statistics on subject characteristics.*** We will summarize subject characteristics across the intervention groups with mean and standard deviation for continuous measures and frequency and proportions for categorical variable using demographic information collected during screening, health status information, food habits data, and compliance to treatment, with special attention to covariates that may influence iron utilization, namely total energy, total protein, and ascorbic acid intakes.

***Planned comparisons.*** Statistical testing is structured to align with the study hypotheses and objectives in a series of planned pairwise comparisons. Comparisons will be made between each of the novel forms of iron and FeSO _4_, administration with multiple micronutrient powder and FeSO _4, _and weekly administration and FeSO _4_. The familywise error rate is set at alpha of 0.05 and is controlled separately for indicators of the safety profile within each research objective. 

***Statistical procedures for hypothesis tests.*** Treatment groups will be analyzed following an intention-to-treat approach. Subjects will not be included in their assigned randomization group for analysis if determined to be nonadherent to the intervention based on compliance and iron status as described under *Interventions*. Responses in malaria infectivity, bacterial proliferation potential, and fecal calprotectin to iron supplementation, quantified as the changes in the respective outcome for each subject over 4 weeks, will be used for comparisons of FeSO _4_ to the other treatment groups. Pairwise comparisons will be made with two-sample t-tests, or linear models allowing adjustment for covariates. Data will be evaluated graphically to assess modeling assumptions including outlier detection, normality, and linearity with covariates. Missing cases in our study will be evaluated separately to assess bias in our sample due to drop outs. Adherence to the intervention will be summarized across all reatment groups based on returned supplement capsules and iron status. Any changes in the primary treatment comparisons after exclusion of nonadherent participants will be reported from secondary per-protocol analysis. All statistical analysis will be performed using SAS v. 9.4 (SAS Institute Inc., Cary, NC) unless otherwise specified.

Specific analysis techniques will be applied to each primary and secondary outcome as follows:

1) Malaria infectivity: Data will be evaluated using the 3D7 sialic acid independent strains of *P. falciparum.* Mean infectivity of erythrocytes will be calculated from experiments performed in duplicate. Four-week change will be quantified for each subject using the logarithm of the ratio of percent parasitized erythrocytes at baseline and post-intervention. Planned comparisons will be tested using two-sample t-tests. Random effects regression will be used to incorporate day-to-day variation and test for batch effects.
2) Bacterial proliferation potential: Bacterial proliferation potential will be evaluated using polynomial growth curve models ^23^; 18-hour growth curves will be fit from mean optical density over three replicates for each subject’s baseline and post-intervention sample. Positive and negative control values on each plate will be used to standardize values to account for day-to-day variation and utilization of different plate readers. Statistical testing will be based on planned comparisons for doubling time during the exponential phase of growth and the maximum level of growth as measured by optical density, calculated from the fitted growth curves. Two-sample t-tests will be used for pairwise group comparisons. We will adjust for multiple tests across the five selected species, using 0.05/5=0.01 type I significance level.
3) Fecal calprotectin: Four-week change in fecal calprotectin will be performed for planned comparisons using two-sample t-tests with log transformation to satisfy normality assumptions.
4) Iron status parameters, redox Stress, gut and systemic inflammation, gut irritation: Changes in continuous-valued clinical and biochemical outcomes will be evaluated using two-sample t-tests. Variable transformations will be used for skewed outcomes, such as number of GI symptoms.
5) Gut microbiome: Diversity measures will be used to compare overall microbiome changes between treatment groups and testing using PERMANOVA. Functional and taxonomic profiling will be used to describe differences between treatment groups. Two-sample tests will be used to test differences in OTU relative abundances and functional predictions, with false discovery rate adjustments for testing across multiple taxa and functions. We will use clustering methods for microbiota data from gut microbiome samples to characterize the association of iron agent and presence of OTU types.

***Bias, confounding, effect modulators.*** Due to the completely randomized design, there will be protection of bias in treatment effects from known and unknown confounding variables. We will also compare subject characteristics across treatment groups and adjust for confounding effects for any variables not balanced through randomization. We are unaware of effect modulators of iron supplementation on *P. falciparum* infectivity and bacterial proliferation. However, outcomes will be evaluated separately at different covariate levels to look for possible modulating effects to be included as interaction terms in our models. Potential effect modulators for fecal calprotectin include age and sex ^35^, and we plan to include them in the models for this outcome.

***Power calculations and sample size.*** The sample size of Phase I and Phase II was determined based on power calculations for each primary outcome as follows:

1) Malaria infectivity: The sample size is based on standard deviation estimates from the *in vitro* assay of malaria infectivity by Clark *et al.* (2014) ^36^. We pooled the estimated variability of percent increase of *P. falciparum* growth analyzed in Clark *et al.* (2014). Using an independent two-sample t-test, an estimated standard deviation 0.202, with a type I error of 0.05, we have at least 90% power to detect a 20% reduction in *P. falciparum* growth using a minimum of 25 subjects per group.
2) Bacterial proliferation potential: Bacterial proliferation potential due to oral iron supplementation was previously studied from a similar *ex vivo* assay ^23^. Between subject estimates of standard deviation were extracted for doubling time during the exponential phase of growth available from three species, *S. aureus*, *E. coli*, and *S. Typhimurium* as 0.2470, (0.530 and 0.212), and (0.813 and 0.071), respectively, with unequal variance estimates used for *E. coli* and *S. Typhimurium* from pre- and post-iron supplementation. Based on two-sample t-tests with type I error at 0.01 (adjusted for planned analysis of five species), and assuming 25 subjects per group, with a minimal detectable difference of 1 hour doubling time, the power is >0.99 for all three species, *S. aureus*, *E. coli*, and *S. Typhimurium*.
3) Fecal calprotectin: Estimates of between subject variation are from subjects with various forms of mild gastrointestinal disease ^30^. Mean fecal calprotectin was reported at 27.6 μg/g in subjects with collagenous colitis, representing mild inflammation, and at 1.9 μg/g in subjects with irritable bowel syndrome, reflecting minimal inflammation. Standard deviation in log concentration of fecal calprotectin used in the sample size calculation is 1.3 µg/g. For our study, in order to detect a 3.5 fold change in fecal calprotectin with 90% power and alpha level of 0.05, we require a minimum of 25 subjects per group. Sample size is based on two-sample t-tests with log-transformed values.

With rolling recruiting and screening, we expect enroll 10–12 subjects/month, randomly assigning them to the intervention treatment groups in blinded fashion. The relatively short length and personalized nature of the intervention will reduce the likelihood of drop outs and allow us to recruit continuously until the recruitment goals are met.

## Methods: Monitoring

### Data and safety monitoring

Ongoing health oversight for subjects in the study will be overseen by study physician. Specific participant complaints will be addressed on an individual basis and appropriate referrals to primary care providers or other health care providers will be made. If problems are reported the subjects will be assessed to determine if the adverse event is potentially study-related and then counseled to seek appropriate assistance. To monitor tolerance of the iron supplements, GI irritation will be assessed by subject interview using a validated GI symptom questionnaire ^34^.

This study presents a minimal risk for subjects; therefore, safety monitoring may be conducted continually by the PI. Despite not requiring an external safety board, this study has an external advisory board who will be involved in the study. The advisory board will review the de-identified data provided to them on a quarterly basis to evaluate the data and monitor subject safety, assessing both harms and benefits. Any reports generated from this advisory board will be submitted to the Tufts Medical Center and Tufts University Health Sciences Campus Institutional Review Board (IRB).

### Trial monitoring

Trial oversight is provided by the study sponsor through the Tufts Medical Center and Tufts University Health Sciences Campus IRB.

## Ethics and dissemination

### Ethics statement

This study has been approved by the IRB at Tufts Medical Center and Tufts University Health Sciences Campus (Phase I IRB #: 12455 and Phase II IRB#: 13341). This study is being conducted in accordance with ICH GCP requirements. Protocol modifications are communicated to the Institutional Review Board at Tufts Medical Center and Tufts University Health Sciences Campus (currently Phase I: Amendment 16). This paper is written following the SPIRIT 2013 guidelines ^37^; a completed SPIRIT checklist is available on Open Science Framework ^21^. This study is registered on ClinicalTrials.gov with the identifier: NCT03212677.

### Dissemination

Key study findings will be presented at relevant international conferences including American Society for Nutrition annual meeting and Micronutrient Forum. The results of the study will be disseminated by submitting manuscript for publication in relevant, peer-reviewed journals. All publications resulting from this research will be published under CC BY 4.0 and made accessible and open immediately in accordance with the Bill and Melinda Gates Foundation open access policy. The data underlying each publication will be combined with the appropriate metadata necessary for reuse and then deposited in the Harvard Dataverse under the Bill & Melinda Gates Foundation Dataverse to ensure that the data is immediately accessible to the greater research community and located in a well-known and easy to locate data repository. Request for use of study data must be approved by the study sponsor and IRB.

### Confidentiality

Participant identifiers will be kept in a separate file from data. Data collection forms will not have participant identifiers. The participant’s identity and records relating to the telephone prescreening and screening information will be kept confidential in locked cabinets at all times at the Volunteer Recruitment Services, and at the MRU, respectively, whether the participant qualifies for the study. All study records for subjects will be kept confidential and the study co-PI, Co-Investigators, Study Physician, Study Coordinators, and Study Statistician will have access to participant identifying information, if needed; otherwise only de-identified data will be transferred to co-investigators. All data will be kept in secure files and locked data cabinets. Identifiers will not be destroyed at any point.

### Study status

For Phase I, screening for enrollment began in June 2017. As of May 2019, 941 subjects have been pre-screened, and 335 subjects have been screened. The first subject baseline (week 0) visit occurred on August 1, 2017 and as of May 2019, 159 subjects have been enrolled in the study. It is anticipated that data collection will be completed by June 2019, with primary outcome analyses and submission for publication completed by August 2019. Current protocol version is April 1, 2019. ****It is anticipated that for Phase II, screening for enrollment will begin June 2019 and data collection will be completed in March 2020. Current protocol version is April 26, 2019. ****

## Discussion

### Rationale for enrolling healthy, iron-replete adults as the population of study

There is strong evidence to support the efficacy of iron supplementation in reducing the risk of iron-deficiency and/or anemia in pre-menopausal women. A recent Cochrane review of ten iron supplementation trials in non-pregnant, pre-menopausal women (12–50 years of age) demonstrated that daily oral iron supplementation reduced the risk of anemia (relative risk: 0.39, 95% CI: 0.25-0.60) ^38^. Despite the efficacy of iron supplementation in reduction of deficiency risk, there are growing concerns of iron supplementation in iron-replete populations. The aforementioned systemic review did not assess potential risks of iron supplementation, including iron overload, oxidative stress, infection and inflammation, as the trials included only physical signs and symptoms associated with supplementation such as GI discomfort ^38^. Based on the available literature, it has been hypothesized that iron-replete individuals may have increased risk of potential adverse effects associated with iron supplementation, particularly in malaria-endemic regions with high levels of inflammation. Most notably, the Pemba sub study, which included 2,413 children undergoing routine iron supplementation, observed an increase in clinical malaria and mortality in iron-replete children ^7^. Although this finding was not statistically significant, it led to the WHO and UNICEF recommending no iron supplementation for young children in malaria-endemic areas and effectively halted programs to address iron deficiency in these areas. The overall goal of the present study is to examine the safety and efficacy of novel forms of iron. Therefore, the rationale for enrolling iron-replete individuals in this study was based on the fact that if adverse effects from iron supplementation were to occur, it would be in this population, rather than an iron-deficient/anemic population.

Regarding the age of our study population, post-menopausal women and age-comparable men in the greater Boston area are being enrolled. It is well recognized that the target population for safe and effective iron supplementation programs is iron-deficient, anemic pre-menopausal women, infants and young children in resource-poor regions. Therefore, the rationale for establishing safety and efficacy in a healthy, older adult population instead of young children was based primarily on ethical considerations. The health of each subject enrolled in this study is monitored weekly, and our study physician, a practicing gastroenterologist, is available for support. While we anticipate the rate of adverse health effects to be low in each of the intervention groups, ethically we wish to first establish safety and efficacy of novel forms of iron supplements in older adults prior to examining the effects in a more vulnerable population such as young children.

### Rationale for daily intervention doses in Phases I and II

Our justification for providing 60 mg iron/day in Phase I is based on the WHO recommendation for daily supplementation for non-anemic, pregnant women with 30–60 mg iron/day ^39^. If the forms of iron are found to produce no adverse effects, or not to worsen adverse effects, at the 60 mg Fe/day dose level, we will carry out the Phase II study in which such forms will be tested at a daily therapeutic dose of 120 mg iron/day against the same outcomes. While it is possible that risks (constipation, diarrhea, black stools and GI irritation) may be greater in Phase II due to the use of this therapeutic dose, the published literature suggests that this may not be the case. The Food and Nutrition Board ^40^ found evidence supporting low adverse effects levels (LOAELs) of FeSO _4_ at doses of 120 mg Fe/day. A more recent meta-analysis of more than 40 clinical trials ^41^ found that the prevalence of gastrointestinal complaints associated with FeSO _4_ administration appear to be idiosyncratic and not related to dose. We anticipate few, if any, complaints associated with either IHAT or Aspiron, as the insolubility of each in the post-duodenal gut would suggest that neither form interacts with the epithelial surfaces.

### Rationale for inclusion of weekly intervention group in Phase I

This study will examine the effects on the “safety profile” of FeSO _4_ administered daily (60 mg iron/day) compared to weekly (420 mg iron administered in one dose/week). In the treatment of iron deficiency, weekly supplementation has been recommended as it presents feasibility and economic advantages to daily supplementation. In various populations, including iron-deficient, anemic lactating women ^42^, non-anemic pregnant women ^43^, non-anemic pregnant women ^44, 45^ and iron-deficient, anemic school-aged children ^46^, it has been demonstrated that a weekly dose of FeSO _4_ can be as effective in improving iron status parameters, but produce lower GI complaints, compared to daily supplementation. Although these studies support the hypothesis that weekly FeSO _4_ supplementation is advantageous over daily supplementation, few studies have directly assessed adverse effects of weekly iron supplementation administration. Inclusion of a weekly FeSO _4_ intervention group in Phase I presents great practical value to support the existing literature in pregnant and early-postpartum women that weekly supplementation of FeSO _4_ is advantageous over daily supplementation.

### Rationale for inclusion of MNP group in Phase I

The primary rationale for use of MNP has been to prevent iron-deficiency and treat iron-deficiency anemia in infants and children ^47^. However, MNP are also administered to other target populations, including pregnant women and may be administered as part of national programs. To address the concerns with iron supplementation in malaria-endemic regions, the World Food Program (WFP) and UNICEF formulated MNP with lower iron content for use in these regions. According to a recent Cochrane review, the use of MNP supplementation reduces both anemia and iron deficiency in young children by 31% and 51%, respectively ^48^. This assessment agrees with the WHO recommendations of utilization of home fortification of foods with MNP ^49^ for improvements in iron status and reduction in iron-deficiency anemia. Although efficacious in the treatment of iron-deficiency anemia, the safety and efficacy of these MNP with and without additional iron has yet to be investigated. In a systemic review examining intervention with MNP in pregnant women, there have been no published studies that have examined the potential benefits or risk of MNP on health outcomes ^50^. Thus, the inclusion of an MNP and FeSO _4_ intervention group in Phase I aims to examine the safety of this recommended, common practice.

### Rationale for the placebo control in Phase I

According to the International Council for Harmonisation of Technical Requirements for Pharmaceuticals for Human Use (ICH) guidelines “Choice of Control Group and Related Issues in Clinical Trials” (ICH E10), the placebo-controlled design acts to control for all potential influences ^51^. As most of our treatment effects focus on the change in our primary outcomes (malaria infectivity, bacterial proliferation and fecal calprotectin), the primary rationale for including a placebo in Phase I is to control for the potential influence in change over time and seasonal effects in these outcomes. The placebo control group will receive the same number of capsules of other treatment groups and contain cornstarch, sourced from Ingredion. Additionally, the ICH guidelines state that “placebo-controlled trials seeks to show a difference between treatments when they are studying effectiveness, but may also seek to show lack of difference in evaluating a safety measurement” ^51^. As this study aims to estimate the “safety profile” between iron supplemented and non-iron supplemented adults, inclusion of a placebo is essential to the study objectives of Phase I. Conclusions on the estimated placebo effect of study participants will be determined from data collected in Phase I.

### Rationale for bacterial species selected for assessing proliferation potential

Five pathogenic bacterial strains were selected for these studies, all serious human pathogens capable of causing bacteremia and spreading through the bloodstream ^52– 56^: two gram-positive ( *A. baumannii* and *S. aureus*), and three gram-negative ( *K. pneumoniae*, *S. typhimurium*, and ExPEC (Extraintestinal Pathogenic *E. coli*)). *A. baumannii* is predominantly an opportunistic pathogen ^52^. *K. pneumonia* causes fatal bacteremia in infants ^53^. *S. typhimurium* is the predominant cause of food poisoning in western countries ^54^. *S. aureus* and ExPEC infections result in high global morbidity and mortality rates ^55, 56^.

## Conclusion

Overall, this double blind, placebo-controlled, randomized controlled trial will robustly test our formal hypotheses and yield useful answers to the primary questions regarding the safety and efficacy of these novel forms of iron supplements. The findings of this study will generate evidence to support development of a modality of providing bioavailable iron that does not or produces less adverse effects in iron-replete individuals. Ultimately, this study aims to contribute to the development of safe and effective forms of supplemental iron to address the global burden of iron deficiency and iron-deficiency anemia.

## Data availability

### Underlying data

No underlying data are associated with this paper.

### Extended data

Open Science Framework: Safe and effective delivery of supplemental iron to healthy volunteers. https://doi.org/10.17605/OSF.IO/U6SAZ ^21^.

The following extended data are available:

- Supplementary File 1. Main Informed Consent Form_Revised May 10, 2019.docx
- Supplementary File 2. Screening ICF_Revised May 10, 2019.docx

### Reporting guidelines

Open Science Framework: SPIRIT checklist for “Safe and effective delivery of supplemental iron to healthy older adults: The double-blind, randomized, placebo-controlled trial protocol of the Safe Iron Study”. https://doi.org/10.17605/OSF.IO/U6SAZ ^21^.

Data are available under the terms of the Creative Commons Zero "No rights reserved" data waiver (CC0 1.0 Public domain dedication).
