# Supplementary material for: Safe and effective delivery of supplemental iron to healthy older adults: The double-blind, randomized, placebo-controlled trial protocol of the Safe Iron Study
Source: Gates Open Res. 2021 Feb 9;3:1510. Originally published 2019 Jul 19. [Version 2] doi: 10.12688/gatesopenres.13039.2 (PMC7890045; doi:10.12688/gatesopenres.13039.2)
Supplement: Peer review history

# Peer review: Reviewer response for version 2

**DOI:** 10.21956/gatesopenres.14450.r30390

**Published:** 2021-02-24

**Authors:** Associated Prof. Sukru Gulec

**Affiliations:** 1 Molecular Nutrition and Human Physiology Laboratory, Food Engineering Department, İzmir Institute of Technology, İzmir, Turkey

The authors indicated explanations for my concern about the manuscript. The manuscript provided valuable information using iron supplementation for humans. Excessive iron might lead to toxicity during consumption. Thus, the manuscript gives us data to use an iron at the safety levels. Moreover, ex vivo studies do not cover the in vivo situation; however, I could understand the study's limitation.

Is the study design appropriate for the research question?

Partly

Is the rationale for, and objectives of, the study clearly described?

Yes

Are sufficient details of the methods provided to allow replication by others?

Yes

Are the datasets clearly presented in a useable and accessible format?

Not applicable

Reviewer Expertise:

Molecular Nutrition

I confirm that I have read this submission and believe that I have an appropriate level of expertise to confirm that it is of an acceptable scientific standard.

# Peer review: Reviewer response for version 2

**DOI:** 10.21956/gatesopenres.14450.r30391

**Published:** 2021-02-17

**Authors:** Richard Hurrell

**Affiliations:** 1 Laboratory of Human Nutrition, Institute of Food, Nutrition and Health, ETH Zurich (Swiss Federal Institute of Technology), Zurich, Switzerland

I am not convinced that the primary ex vivo outcomes will provide useful new information. However, I approve the study for the secondary outcomes of NTBI, iron status, and potential adverse side effects. The secondary outcomes will provide important new information.

Is the study design appropriate for the research question?

Partly

Is the rationale for, and objectives of, the study clearly described?

Yes

Are sufficient details of the methods provided to allow replication by others?

Yes

Are the datasets clearly presented in a useable and accessible format?

Not applicable

Reviewer Expertise:

NA

I confirm that I have read this submission and believe that I have an appropriate level of expertise to confirm that it is of an acceptable scientific standard.

# Peer review: Reviewer response for version 2

**DOI:** 10.21956/gatesopenres.14450.r30389

**Published:** 2021-02-17

**Authors:** Nana Gletsu-Miller

**Affiliations:** 1 Department of Applied Health Science, School of Public Health, Indiana University Bloomington, Bloomington, IN, USA

This is a good protocol paper that describes a RCT that is rigorous in design and the detail provided ensures that transparency and reproducibility have been considered. Findings should be impactful for prevention or treatment of iron deficiency anemia. My original review was that the manuscript should be accepted with minimal revisions. In the revised manuscript, the authors addressed most of the comments that I suggested. In particular, I now understand their decision to use post-menopausal women as the study population even though children and pre-menopausal women are the population of interest. I defer to the other reviewers to assess the authors' responses to their comments.

Is the study design appropriate for the research question?

Partly

Is the rationale for, and objectives of, the study clearly described?

Partly

Are sufficient details of the methods provided to allow replication by others?

Yes

Are the datasets clearly presented in a useable and accessible format?

Not applicable

Reviewer Expertise:

Nutrition science, iron deficiency, bariatric surgery

I confirm that I have read this submission and believe that I have an appropriate level of expertise to confirm that it is of an acceptable scientific standard.

# Peer review: Reviewer response for version 1

**DOI:** 10.21956/gatesopenres.14152.r29204

**Published:** 2020-09-03

**Authors:** Associated Prof. Sukru Gulec

**Affiliations:** 1 Molecular Nutrition and Human Physiology Laboratory, Food Engineering Department, İzmir Institute of Technology, İzmir, Turkey

The study aims to develop new iron supplement forms (IHAT and aspiron) and test their physiological features comparing to the FeSo _4_ form. The study contained two phases to compare two different Fe supplementation amounts, including 60mg/day and 120mg/day for four weeks. The older adults living in the Boston area were selected as the study group. The hypothesis was tested by a randomized, double-blinded, placebo-controlled study.

 The main concern about the manuscript:

- The efficacy of IHAT and aspiron were tested on the people from the Boston area. However, one of the hypotheses of the study was malaria infectivity that is the target for the Tanzania population. We see two different populations into the study, so how do the populations model each other for iron supplementation and malaria infectivity experiments? Furthermore, the risk of iron deficiency also commonly seen in young children/childbearing ages and iron supplamentation is also essential for them to reduce iron deficiency anemia. Why did not researchers also focus on those groups?
- The researchers compared daily (60mg iron/day) and weekly (420mg iron/week) iron administration. How do they relate 420 mg/week iron administration to 60mg iron/day in terms of physiological consequences? The older people have a risk for iron overload, so 420 mg iron/day might have a negative effect on them. The also 120 mg iron/day supplementation brings the same risk for the older population.
- The inflammation (mainly systemic)  and iron metabolism are closely related via, for instance, IL-6 and hepcidin. Thus, systemic inflammatory markers (IL-6, TNFa) might be important to compare iron supplementation efficacy on the study groups. Moreover, their basal levels must also be considered in the study groups.  
- The environmental area might affect the health status of people in Tanzania, and they might have very different physiological situations (inflammatory levels in the body) than the study group. As a result, this might cause a different response to iron metabolism. Thus, iron supplementation strategies might not resemble the real situation in Tanzania.
- How do ex vivo experiments cover the similar responses that are Tanzania population? The authors indicated ‘…increased severity of clinical malaria among children in Tanzania effectively halted efforts to address iron deficiency in malaria-endemic areas. Lack of a safe and effective treatment leaves large numbers of children iron deficient, many with associated anemia…’. They stated malaria problems in children, but the investigation was performed in older adults. How do researchers conclude that?

Is the study design appropriate for the research question?

Partly

Is the rationale for, and objectives of, the study clearly described?

Yes

Are sufficient details of the methods provided to allow replication by others?

Yes

Are the datasets clearly presented in a useable and accessible format?

Not applicable

Reviewer Expertise:

Molecular Nutrition

I confirm that I have read this submission and believe that I have an appropriate level of expertise to confirm that it is of an acceptable scientific standard, however I have significant reservations, as outlined above.

# Peer review: Reviewer response for version 1

**DOI:** 10.21956/gatesopenres.14152.r29198

**Published:** 2020-08-10

**Authors:** Nana Gletsu-Miller

**Affiliations:** 1 Department of Applied Health Science, School of Public Health, Indiana University Bloomington, Bloomington, IN, USA

This manuscript describes the rationale and design of a clinical trial to evaluate iron supplementation regimens that are safe and effective. The premise of the study is the oral iron supplementation is recommended for prevention and treatment of iron deficiency, one of the most widespread nutritional disorders worldwide. However, oral iron supplementation is not often effective because the gastrointestinal side effects prevent people from complying with the regimens. Other side effects of oral iron supplementation are systemic oxidative stress and growth enhancement of unfavorable gut and systemic bacteria. The investigators designed the study to address the need for oral iron supplemental regimens that are safe and effective. Ferrous sulfate serves as the standard formulation and it is compared to placebo (corn starch), as well as to two novel iron formulations, providing iron weekly rather than daily, and providing multiple micronutrients along with the iron. The study was designed to have two phases, both of which are well underway and may already be completed by the time of this review. Thus, while there are several strengths of the study, weaknesses of the study design are not modifiable, which is a major criticism of this review. 

 Strengths: 

- The study is rigorously designed to test various hypotheses that the experimental regimens are as safe as ferrous sulfate, but have better safety profiles which are assessed as primary and secondary outcomes (e.g. malaria infectivity, bacterial proliferation, gut inflammation, oxidative stress, gut microbiota profile). This is a randomized, double-blinded, controlled, comparative effectiveness intervention.
- The design is innovative as only the safe and effective regimens from phase 1 will be used in phase 2. Phase 1 will test lower doses of the regimen and Phase 2 will test a dose that may be used therapeutically.
- The description of the design and conduct of the study is thorough allowing for transparency and reproducibility.
- The study is well underway, which demonstrates the feasibility of recruitment and conduct of the protocol.

Major weaknesses:

- Characterizing the phase 1 dose, 60 mg Fe/day as low is debatable, since it is several times higher than the RDA (18 mg/day Fe) and some treatment guidelines recommend this as a treatment dose (e.g., Killip et al, Am Fam Phys, 2007). 
- Choosing post-menopausal women or men for the study population is not well-justified, given this population is least likely to develop iron deficiency, reducing the generalizability of the study’s findings. The authors state correctly that women of childbearing age are most at risk for iron deficiency so why not test the regimens in this population?
- Related to the previous point, since post-menopausal women and men are most at risk for iron overload, providing 60mg Fe/day or 120 mg Fe/day for 4 weeks to iron-replete members individuals seems risky. The tolerable upper limit for this population is 45 mg Fe/day.
- The statistical analysis chooses an “as treated approach”. Since the high doses may lead to poor adherence to the regimens, it seems that an intention to treat analysis would be less prone to bias.
- Adding the multiple micronutrient to the iron supplement as a treatment regimen is not well justified and seems outside of the scope of the study, per the study’s hypothesis: “…examining the safety and efficacy of these novel forms of iron supplement in iron-replete older adults”. Similarly, adding an arm to test the weekly versus daily dose of ferrous sulfate is not consistent with the objectives of the study.
- Information on Aspiron regarding NTBI is not complete, should include study - Bries Bries, Curr Develop Nutr, 2019. 

Minor comments:

- Statements requiring citations:
  - Introduction – (i.e., 5-20 fold effective levels of heme-iron in foods).
  - Discussion – Rationale for daily intervention doses – WHO recommendation for daily supplementation for non-anemic pregnant women.
- Typos in Abstract and throughout document.

Is the study design appropriate for the research question?

Partly

Is the rationale for, and objectives of, the study clearly described?

Partly

Are sufficient details of the methods provided to allow replication by others?

Yes

Are the datasets clearly presented in a useable and accessible format?

Not applicable

Reviewer Expertise:

Nutrition science, iron deficiency, bariatric surgery

I confirm that I have read this submission and believe that I have an appropriate level of expertise to confirm that it is of an acceptable scientific standard, however I have significant reservations, as outlined above.

# Peer review: Reviewer response for version 1

**DOI:** 10.21956/gatesopenres.14152.r28311

**Published:** 2019-12-09

**Authors:** Richard Hurrell

**Affiliations:** 1 Laboratory of Human Nutrition, Institute of Food, Nutrition and Health, ETH Zurich (Swiss Federal Institute of Technology), Zurich, Switzerland

**Overview:**

 The Safe Iron Study could potentially provide new information that would help prevent the serious adverse events observed with iron supplementation in low-and-middle income countries (LMIC) where poor hygiene and widespread infections are common, and malaria frequently endemic. These serious adverse events, as a result of iron supplementation, need urgent attention as they have restricted iron interventions especially in malaria endemic areas.

**Aim of Study:**

 The aim of the Safe Iron Study is to find a way of providing bioavailable iron, in the form of supplements, to iron deficient populations in LMIC without causing adverse side effects, including infectious diarrhea, increased susceptibility to malaria infection, increased inflammation, and detrimental changes to the gut microbiome. It is hoped that this can be achieved by using iron hydroxy adipate tartrate (IHAT) or Aspiron (dried iron-enriched Aspergillus Oryzae biomass) instead of ferrous sulfate as the iron compound for the iron supplements. These novel iron compounds have both been reported to have equivalent iron absorption to ferrous sulfate but to be absorbed more slowly, and they are predicted to have less adverse side effects. More specifically, they are expected to generate less non-transferrin bound iron (NTBI) than ferrous sulfate, and to be less well-metabolized by pathogenic organisms in the hind gut, and thus increasing pathogen numbers in the gut microbiome to a lower extent. 

**Proposed Study Protocol:**

 The safe Iron Study will compare iron supplements based on IHAT, and supplements based on Aspiron, with ferrous sulfate supplements, given to older (50-80 years old) iron replete adults living in the Boston area. Two feeding studies are planned, (Phase 1 and Phase 2) in which the iron replete older adults are provided with 60mg Fe supplements daily for 4 weeks (Phase 1) or 120mg Fe supplements daily for 4 weeks (Phase 2). Phase 1 has an additional 3 groups. These are a placebo group, a group receiving 60mg Fe as ferrous sulfate plus a micronutrient powder once per day, and a group receiving 420 mg Fe as ferrous sulfate once a week. The additional groups in Phase 1 provide data on a control group with no iron supplement (which can be also be used in Phase 2), and allow an evaluation of whether providing iron within a micronutrient powder, or as a single high dose supplement once a week, changes the observed response to iron supplementation.

 In Phase 1 and Phase 2, blood and stool samples are taken at baseline and at 4 weeks. The primary outcomes of the study are malarial infectivity (ex vivo) in the presence of host erythrocytes, bacterial proliferation (ex vivo) in the presence of host plasma, and intestinal inflammation as measured by fecal calprotectin. The secondary outcomes are iron status as measured using a range of iron status biomarkers parameters, NTBI and systemic inflammation biomarkers, all measured in blood; and fecal iron, intestinal inflammation biomarkers and changes in the gut microbiome, all measured in stool.

**Comments on the study design and methodology:**

 While I am in strong support of the need for further research that enables iron supplements to be given safely in LMIC, and in support of much of the proposed program, I am not in complete agreement with the need to study the increased susceptibility to malaria infection ex vivo in the host blood, nor do I see much usefulness in measuring bacterial proliferation ex vivo in host plasma. Both measurements are primary outcomes of this study.

  

1. In relation to the malarial infection, the increased mortality and morbidity reported after iron supplementation (Sazawal *et al.*, 2006 ^1^) appear to be due to an intensification of the malarial infection, leading to more cerebral malaria and bacteremia, rather than to an increase in the number of malaria infections. The underlying cause of the intensification of a malaria infection is thought to be the formation NTBI in the blood serum on absorption of the relatively large dose of iron in the supplements (Hurrell, 2010 ^2^ and Hurrell, 2011 ^3^). The likely explanation for NTBI formation is that the rate of iron influx into the plasma from high-dose oral supplements exceeds the rate of iron binding to transferrin. It is proposed that NTBI increases the severity of malarial infections by increasing the sequestration of malaria-infected erythrocytes in the capillaries of the brain and intestine (Kartikarsari *et al.*, 2006 ^4^), leading to more cerebral malaria and a breach of the intestinal barrier leading to bacteremia. At the same time, high iron doses in supplements can stimulate the growth of pathogenic bacteria in the stool, further increasing the potential for bacteremia, which is frequently reported in children with severe malaria (Oundo *et al.*, 2002 ^5^). 
  While the usefulness of measuring malaria infectivity ex vivo as a primary outcome can be questioned, the Safe Iron Study is measuring NTBI as a secondary outcome so will compare the iron supplements in relation to their ability to increase the intensity of the malaria infection. NTBI will be measured at baseline and 2h after supplement ingestion. Results from the NTBI measurements are perhaps the most important new outcome from the study and could be upgraded from a secondary outcome to a primary outcome of the study. 
2. The second primary outcome of the Safe Iron Study is bacterial proliferation (ex vivo) in the presence of host plasma. The reason for these studies is presumably to compare the risk for bacteremia during malaria infection after ferrous sulfate supplementation with that of the novel iron compounds which are absorbed more slowly. The serum will be tested at baseline and 2h after supplement ingestion. At the 2h time point, the serum taken after ferrous sulfate ingestion would be expected to be higher in iron than the serum taken after ingestion of the more slowly absorbed novel iron compounds. The 2h serum after ferrous sulfate ingestion therefore would be expected to increase pathogen growth to a greater extent. 
  While the risk of bacteremia may depend to some extent on the iron concentration of the blood plasma, the greater risks for bacteremia appear to be the permeability of the gut mucosa (depending on NTBI formation) and the number of pathogens present in the gut microbiome. Iron supplementation to children living areas of widespread infection has been reported to increase pathogen numbers (Zimmermann *et al.*, 2010 ^6^) in the stool. In children with severe malaria, it is possible that these pathogens enter the blood stream via the damaged capillaries in the intestinal villi. The pathogens could presumably enter the blood stream at any time, so the ideal times within the Safe Iron Study to take sera for bacterial proliferation measurements are not easy to select. As iron absorption from ferrous sulfate, IHAT and Aspiron is reported to be similar, the total amount of iron entering the plasma over a 24h period between supplement ingestion would be similar, but the pattern of entry would be different.
  Increased pathogen numbers in the gut microbiome of children living in areas of poor hygiene and widespread infections is clearly a major safety issue in relation to iron supplementation, and is a major risk for bacteremia during malaria infection, in addition to being a risk for infectious diarrhea. The Safe Iron Study proposes to measure the gut microbiome in older, iron replete adults in Boston after ingestion of IHAT and Aspiron supplements. The choice of subjects, and the choice of a hygienic environment in which to make the study, can be explained by a need for absolute safety. However, ferrous sulfate supplements are still regularly administered in Sub Saharan Africa to women living in areas of poor hygiene and widespread infections without the serious side effects reported in children, and IHAT and Aspiron administration has caused no serious negative side effects in women living in industrialized countries. Feeding the compounds in Boston, will lead to new information concerning their influence on the gut microbiome. However, it will add little to our knowledge regarding potential adverse events resulting from iron supplementation in countries with poor hygiene and widespread infections. There would appear to be no safety concerns to making a similar Safe Iron Study in young women in sub Saharan Africa study so as to compare the gut microbiome after IHAT, Aspiron and ferrous sulfate supplementation, in a region where ferrous sulfate supplementation is commonly practiced. Such a study would provide vital new information and would be the major first step the evaluation of the safety of IHAT and Aspiron. 
3. Another concern relates to the choice of iron replete older adults as the study subjects. This is based on the suggestion from the Pemba study that the serious adverse events occurring with iron supplementation in malaria endemic areas are observed mostly in iron replete children. This “finding” however was not statistically significant (so is really only a suggestion), it has not been confirmed in further studies, and there is no clear mechanism to explain why iron replete subjects have more adverse events. Iron absorption would be extremely low in iron replete subjects with malarial infection and inflammation, and a mechanism for serious adverse events that is linked to absorbed iron would be less likely. Young women of marginal or low iron status may be more appropriate subjects than older iron replete adults as they would be expected to exhibit a higher iron absorption. This would facilitate finding differences between iron compounds in relation to NTBI, systemic inflammation biomarkers and iron status biomarkers. Potential side effects of iron supplementation of women in high income countries and also LMIC, are well known and are not considered a safety issue. 
4. The measurement of fecal calprotectin as a primary outcome will generate important new information on IHAT and Aspiron in older adults in a hygienic environment. A similar study may be needed in adults living in a less hygienic environment. An increase in fecal calprotectin has already been reported with iron fortification in children in the Cote d’Ivoire (Zimmermann *et al.*, 2010 ^6^).
5. The measurement of iron status biomarkers in the blood should confirm the high bioavailability of IHAT and Aspiron, whereas the measurement of systemic inflammation biomarkers in the blood and fecal iron should generate useful new information. 

 

**Conclusion:**

 The Safe Iron Study aims to find a way to provide supplements of bioavailable iron to populations living in areas of poor hygiene and widespread infection without increasing infectious diarrhea, the susceptibility to malaria infection, inflammation, and detrimental changes to the gut microbiome. The approach to compare daily supplements of ferrous sulfate with supplements of IHAT and Aspiron, in iron replete older adults living in Boston in the United States may be overcautious and will provide little new information on the safety of IHAT and Aspiron with respect to infectious diarrhea, inflammation or changes gut microbiome. Important new results on NTBI should help judge their ability to intensify malarial infections, and iron status measurements should confirm their bioavailability. The primary outcomes of malaria infectivity in the presence of host erythrocytes and pathogen proliferation in the presence of host plasma are judged to be of limited usefulness. 

 Increased morbidity and mortality with iron supplementation of malaria infected children appear to be due to the formation of NTBI. It is proposed that NTBI increases the severity of malarial infections by increasing the sequestration of malaria-infected erythrocytes in the capillaries of the brain and intestine leading to more cerebral malaria and a breach of the intestinal barrier leading to bacteremia. At the same time, high iron doses in supplements can stimulate the growth of pathogenic bacteria in the stool, further increasing the potential for bacteremia. An important first step in the evaluation of IHAT and Aspiron would be to compare their influence on pathogen numbers in the gut microbiome of young women living in Sub Saharan Africa, with the influence of ferrous sulfate.

Is the study design appropriate for the research question?

Partly

Is the rationale for, and objectives of, the study clearly described?

Yes

Are sufficient details of the methods provided to allow replication by others?

Yes

Are the datasets clearly presented in a useable and accessible format?

Not applicable

Reviewer Expertise:

micronutrient interventions, iron and malaria, iron status

I confirm that I have read this submission and believe that I have an appropriate level of expertise to confirm that it is of an acceptable scientific standard, however I have significant reservations, as outlined above.

## References

[1] : Effects of routine prophylactic supplementation with iron and folic acid on admission to hospital and mortality in preschool children in a high malaria transmission setting: community-based, randomised, placebo-controlled trial. *The Lancet*.2006;367(9505) : 10.1016/S0140-6736(06)67962-2 133-143 10.1016/S0140-6736(06)67962-2 16413877

[2] : Iron and malaria: absorption, efficacy and safety. *Int J Vitam Nutr Res*.2010;80(4-5) : 10.1024/0300-9831/a000035 279-92 10.1024/0300-9831/a000035 21462111

[3] : Safety and efficacy of iron supplements in malaria-endemic areas. *Ann Nutr Metab*.2011;59(1) : 10.1159/000332140 64-6 10.1159/000332140 22123642PMC7265417

[4] : Endothelial activation and induction of monocyte adhesion by nontransferrin-bound iron present in human sera. *FASEB J*.2006;20(2) : 10.1096/fj.05-4700fje 353-5 10.1096/fj.05-4700fje 16368718

[5] : Non-typhi salmonella in children with severe malaria. *East Afr Med J*.2002;79(12) : 10.4314/eamj.v79i12.8670 633-9 10.4314/eamj.v79i12.8670 12678446

[6] : The effects of iron fortification on the gut microbiota in African children: a randomized controlled trial in Cote d'Ivoire. *Am J Clin Nutr*.2010;92(6) : 10.3945/ajcn.110.004564 1406-15 10.3945/ajcn.110.004564 20962160
